# Supplementary material for: Scenario-feature identification from online reviews based on BERT
Source: PeerJ Comput Sci. 2023 May 22;9:e1398. doi: 10.7717/peerj-cs.1398 (PMC10280460; doi:10.7717/peerj-cs.1398)
Supplement: Supplemental Information 1 [file peerj-cs-09-1398-s001.zip › Data and processing codes/codebook.docx]

**Directory**

Build Co occurrence Matrix

CoMatrix.py

CoMatrixProcess(1).py

MI calculation

MIS.py

Pre-processing

zh_wiki.py

langconv.py

Splitting sentences.py

participle.py

Remove stop words.py

Word filtering.py

word frequency count.py

Word Marking.py

Word vector.py

Calculate semantic similarity.py

Text classification

ArrangDataSet.py

Calculation of recall rate, etc.py

Forecast New Data Labels.py

Model.py
